# RE-AIM (Reach, Effectiveness, Adoption, Implementation, and Maintenance) Evaluation of the Use of Activity Trackers in the Clinical Care of Adults Diagnosed With a Chronic Disease: Integrative Systematic Review

**DOI:** 10.2196/44919

**Published:** 2023-11-13

**Authors:** William Hodgson, Alison Kirk, Marilyn Lennon, Xanne Janssen, Eilidh Russell, Carolina Wani, Dina Eskandarani

**Affiliations:** 1 School of Psychological Sciences and Health Department of Physical Activity for Health University of Strathclyde Glasgow United Kingdom; 2 Department of Computer and Information Sciences University of Strathclyde Glasgow United Kingdom

**Keywords:** activity trackers, clinical care, physical activity, sedentary behavior, adults, chronic diseases, Reach, Effectiveness, Adoption, Implementation, and Maintenance, RE-AIM, mortality, sedentary lifestyle, intervention, mobile phone

## Abstract

**Background:**

Chronic diseases are a leading cause of adult mortality, accounting for 41 million deaths globally each year. Low levels of physical activity and sedentary behavior are major risk factors for adults to develop a chronic disease. Physical activity interventions can help support patients in clinical care to be more active. Commercial activity trackers that can measure daily steps, physical activity intensity, sedentary behavior, and distance moved are being more frequently used within health-related interventions. The RE-AIM (Reach, Effectiveness, Adoption, Implementation, and Maintenance) framework is a planning and evaluation approach to explore the reach, effectiveness, adoption, implementation, and maintenance of interventions.

**Objective:**

The objective of this study is to conduct an integrative systematic review and report the 5 main RE-AIM dimensions in interventions that used activity trackers in clinical care to improve physical activity or reduce sedentary behavior in adults diagnosed with chronic diseases.

**Methods:**

A search strategy and study protocol were developed and registered on the PROSPERO platform. Inclusion criteria included adults (18 years and older) diagnosed with a chronic disease and have used an activity tracker within their clinical care. Searches of 10 databases and gray literature were conducted, and qualitative, quantitative, and mixed methods studies were included. Screening was undertaken by more than 1 researcher to reduce the risk of bias. After screening, the final studies were analyzed using a RE-AIM framework data extraction evaluation tool. This tool assisted in identifying the 28 RE-AIM indicators within the studies and linked them to the 5 main RE-AIM dimensions.

**Results:**

The initial search identified 4585 potential studies. After a title and abstract review followed by full-text screening, 15 studies were identified for data extraction. The analysis of the extracted data found that the RE-AIM dimensions of adoption (n=1, 7% of studies) and maintenance (n=2, 13% of studies) were underreported. The use of qualitative thematic analysis to understand the individual RE-AIM dimensions was also underreported and only used in 3 of the studies. Two studies used qualitative analysis to explore the effectiveness of the project, while 1 study used thematic analysis to understand the implementation of an intervention.

**Conclusions:**

Further research is required in the use of activity trackers to support patients to lead a more active lifestyle. Such studies should consider using the RE-AIM framework at the planning stage with a greater focus on the dimensions of adoption and maintenance and using qualitative methods to understand the main RE-AIM dimensions within their design. These results should form the basis for establishing long-term interventions in clinical care.

**Trial Registration:**

PROSPERO CRD42022319635; https://www.crd.york.ac.uk/prospero/display_record.php?RecordID=319635

## Introduction

Noncommunicable diseases, also referred to as chronic diseases, are a major cause of premature death. They include cardiovascular disease, chronic respiratory disease, cancer, and diabetes mellitus. It is estimated that globally 41 million people die each year from chronic diseases, and this accounts for 71% of all deaths worldwide [1]. Major risk factors for the development of chronic diseases are low levels of physical activity and high levels of sedentary behavior [2]. It is estimated that globally 23% of adults are failing to achieve the recommended physical activity guidelines set by the World Health Organization [1]. The adult guidelines recommend 150 minutes of moderate to vigorous intensity physical activity per week, to undertake muscle exercises at least 2 days per week, and to reduce sedentary time [3].

Physical activity interventions have been developed in an effort to encourage individuals to be more active within both primary health care and community-based settings [4]. Activity trackers, such as Fitbit (Fitbit), Polar (Polar Electro), and Garmin (Garmin Ltd), which can monitor and provide feedback on steps, physical activity intensity, distance moved, and sedentary behavior, can support patients in leading a more active lifestyle and could be a cost-effective public health intervention [5].

During test and retest trials, activity trackers and pedometers were found to be valid and reliable methods of measuring steps (Misfit Shine, Withings Pulse, Fitbit Zip, and Digiwalker) [6]. Fitbit’s activity trackers (Ultra, One, Zip, Flex, Force, Charge, Charge HR, and Surge), for example, have been shown to have high interdevice reliability in respect of distance moved, steps, and energy expenditure [7]. A recent systematic review highlighted that the use of activity trackers by patients diagnosed with a chronic disease (chronic respiratory disease, type 2 diabetes, and cardiovascular disease) would significantly increase the number of daily steps taken (2123) and decrease a patient’s systolic blood pressure (–3.79 mm Hg), waist circumference (–0.99 cm), and low-density lipoprotein cholesterol concentration (–5.70 mg/dL) [8].

Interventions involving changes in lifestyle such as increased physical activity, reduction in sedentary time, and weight loss can significantly reduce the risk of adults developing chronic diseases and related health complications. Weight loss programs involving dietary and physical activity education coupled with the use of an activity tracker have been shown to significantly reduce the weight of participants [9].

While many studies involving public health interventions focus on effect and efficacy, these fail to fully evaluate the impact of such research when applied to real-world settings. The RE-AIM (Reach, Effectiveness, Adoption, Implementation, and Maintenance) framework was developed as both a planning and evaluation tool aimed at understanding the full impact of a public health intervention and how this could best be introduced into patient health care [10]. The framework comprises 5 main dimensions: reach, effectiveness, adoption, implementation, and maintenance. Reach seeks to measure the proportion of the targeted population who took part in the intervention. Effectiveness aims to understand the success of the intervention. Adoption covers the number of settings, practices, and plans that will engage with the intervention. Implementation looks at how the intervention was applied in practice. Maintenance seeks to understand the long-term viability of the project [10]. Each dimension should be considered when planning or evaluating a public health intervention. The main dimensions are subsequently subdivided into indicators that facilitate a detailed evaluation of each dimension [10]. Though each dimension should be considered at the planning stage of an intervention, it is not imperative to report in detail all components. Greater focus on those applicable to the individual study or clinical organization has been shown to improve the translation from research into practical health care, which has proved to be a barrier in the past [10].

The aim of this integrative systematic review was to comprehensively explore the reporting of the reach, effectiveness, adoption, implementation, and maintenance of using activity trackers in clinical care to support physical activity or reduce sedentary behavior in adults diagnosed with chronic diseases. The integrative methodology allows for the review of quantitative, qualitative, and mixed methods research while developing a comprehensive understanding of the RE-AIM elements reported and the particular health care problem under review [11].

## Methods

### Study Design

The protocol for this integrative systematic review was registered in the PROSPERO international prospective register of systematic reviews on March 23, 2022 (CRD42022319635). The review was undertaken in accordance with the PRISMA (Preferred Reporting Items for Systematic Reviews and Meta-Analyses) 2020 statement [12]. All published papers in English between 2015 and 2022 were included.

### Eligibility Criteria

Textbox 1 provides a summary of the inclusion criteria using PICOS (population, intervention type, comparisons, outcomes of interest, and study type) for quantitative studies and SPIDER (sample, phenomenon of interest, study design, evaluation, and research type) for qualitative studies.

**Inclusion criteria based on PICOS (population, intervention type, comparisons, outcomes of interest, and study type) and SPIDER (sample, phenomenon of interest, study design, evaluation, and research type).**

**PICOS criteria**

- Population: Adults (18 years and older) diagnosed with a chronic disease
- Intervention type: Individual or combined use of an activity tracker within outpatient clinical care
- Comparisons: All control or comparison groups
- Outcomes of interest: RE-AIM (Reach, Effectiveness, Adoption, Implementation, and Maintenance) dimensions of using an activity tracker within outpatient clinical care
- Study type: Intervention or experimental and observational

**SPIDER criteria**

- Sample: Adults (18 years and older) diagnosed with a chronic disease and adult (18 years and older) health care professionals working within a clinical capacity
- Phenomenon of interest: Experiences of using an activity tracker within outpatient clinical care
- Study design: Qualitative and mixed methods
- Evaluation: RE-AIM dimensions of using an activity tracker within outpatient clinical care
- Research type: Qualitative and mixed methods (qualitative and quantitative)

### Search Strategy and Information Sources

A detailed search strategy was developed, with guidance from a qualified university librarian, around the core themes and RE-AIM dimensions. The key search words are displayed in Table 1. These were developed around the key original terms: “activity trackers,” “clinical care,” “physical activity,” “sedentary behaviour,” “adults,” “chronic diseases,” and “RE-AIM.”

**Table 1:** Key search words focusing on the core themes and RE-AIM^a^ dimensions.

Original term	Broader terms
Activity trackers	Activity monitor, wearable technology, wearable device, eHealth, mHealth, fitness tracker, fitness device, digital intervention, digital tracker, digital monitor, digital device, wearable activity tracker, pedometer, accelerometer, and step counter
Clinical care	Health care, healthcare, primary care, outpatient, out-patient, clinical practice, public health, clinical, care, and health promotion
Physical activity	Physical fitness, active lifestyles, fitness, physical health, activity, exercise, and intervention
Sedentary behaviour	Sedentary, sedentary time, sitting time, sitting, sitting behaviour, screen time, screen based, chair based, deskbound, physical inactivity, inactive lifestyle, and lack of activity
Adult	Adult and adults
Chronic diseases	Chronic illness, noncommunicable disease, noncommunicable, non-communicable, chronic disease, chronic, illness, and disease
RE-AIM	Validity, external validity, behaviour change, policy change, community change, participation, quality of life, reach, influences, effectiveness, success, usefulness, efficacy, adoption, acceptance, maintenance, preservation, acceptability, rate, appraise, analysis, implement*, implementation, and deliver

^a^RE-AIM: Reach, Effectiveness, Adoption, Implementation, and Maintenance.

The study searches were undertaken on the computer-based databases: Web of Science—Core Collection (all editions; Clarivate), MEDLINE (ProQuest; ProQuest), ACM (Digital Library; ACM Digital Library), APA PsycINFO (EBSCO), Cochrane Library (Cochrane), MEDLINE (Ovid, Embase, and Embase Classic; MEDLINE), and gray literature (GreyNet; GreyNet International), CORE (Core), OAIster and WorldCat.org (OCLC), and Open Access Thesis and Dissertations (OATD).

### Screening Process

Papers identified during the search phase were initially uploaded onto EndNote (Clarivate). Duplicates were identified and removed. Papers were subsequently uploaded onto the Rayyan (Rayyan Systems Inc) collaborative systematic review screening system. All remaining papers were screened on the Rayyan system by 4 authors (WH, ER, CW, and DE) based on the topic, title, and abstract. Last, a full-text screening of the remaining papers was conducted by 2 authors (WH and ER). Conflicts were resolved at each stage through discussion between the screening authors without the need for an independent review.

### Data Extraction

The final papers identified for review were uploaded onto a validated RE-AIM extraction Microsoft Excel spreadsheet (Microsoft Corporation; Multimedia Appendix 1). This spreadsheet was developed by combining 2 validated RE-AIM extraction tools, and it contains details of each paper, the 5 main RE-AIM dimensions (reach, effectiveness, adoption, implementation, and maintenance), and 28 RE-AIM indicators [11-14]. The identified papers were screened, and data were extracted by the main author (WH). This data extraction was quality-checked by 3 authors (AK, XJ, and ML), who reviewed 2 separate papers each. Conflicts were resolved through discussion between all data extraction authors. The PRISMA checklist was followed throughout [12] (Multimedia Appendix 2).

### Data Items

Basic participant demographic details were extracted from each paper including number of participants, mean age, and gender. Each of the 5 main RE-AIM dimensions is supported by identifiable indicators (reach: n=7, effectiveness: n=6, adoption: n=6, implementation: n=5, and maintenance: n=4) [11-14]. The main RE-AIM dimensions and supporting indicators are displayed in Textbox 2.

**Main RE-AIM (Reach, Effectiveness, Adoption, Implementation, and Maintenance) dimensions and supporting indicators.**

**Reach**

- Method used to identify the target population
- Inclusion criteria
- Exclusion criteria
- Use of qualitative methods to understand reach or recruitment
- Sample size
- Participation rate
- Sample representatives

**Effectiveness**

- Assessment of the effect on outcomes at the shortest assessment point
- Imputation procedures reported (how missing data are processed)
- The presence of quality-of-life measure
- Effects at the longest follow-up
- Use of qualitative methods to understand outcomes
- Percent attrition or dropout rate

**Adoption**

- Method of identifying target agent
- Level of expertise of delivery agents
- Inclusion and exclusion criteria for target agent
- The adoption rate
- Comparison of settings or participants of adoption versus nonadoption settings
- Use of qualitative methods to understand either adoption at the setting level or staff participation

**Implementation**

- The intervention type (individual component vs multicomponent)
- Intensity (components of intervention)
- The extent the protocol was delivered as intended
- A measure of cost
- Use of qualitative methods to understand the implementation of the study

**Maintenance**

- Was an individual’s behavior assessed at least 6 months following completion of the intervention
- Is the program still in place
- Was the program modified
- Use of qualitative methods to understand the long-term effects

Each identified journal paper was screened as previously described, and the reporting of each RE-AIM indicator was recorded on the RE-AIM data extraction sheet. The percentage of papers reporting the individual indicator was calculated.

### Study Risk of Bias Assessment and Reporting Bias Assessment

As the RE-AIM framework is an evaluation tool designed to identify and focus on the reporting of recommended dimensions and indicators within interventions, no additional risk of bias assessment was conducted [15].

### Outcome Measures

The total percentage of individual RE-AIM indicators reported from all journal papers was the main outcome measure.

## Results

### Overview

**Figure 1:**
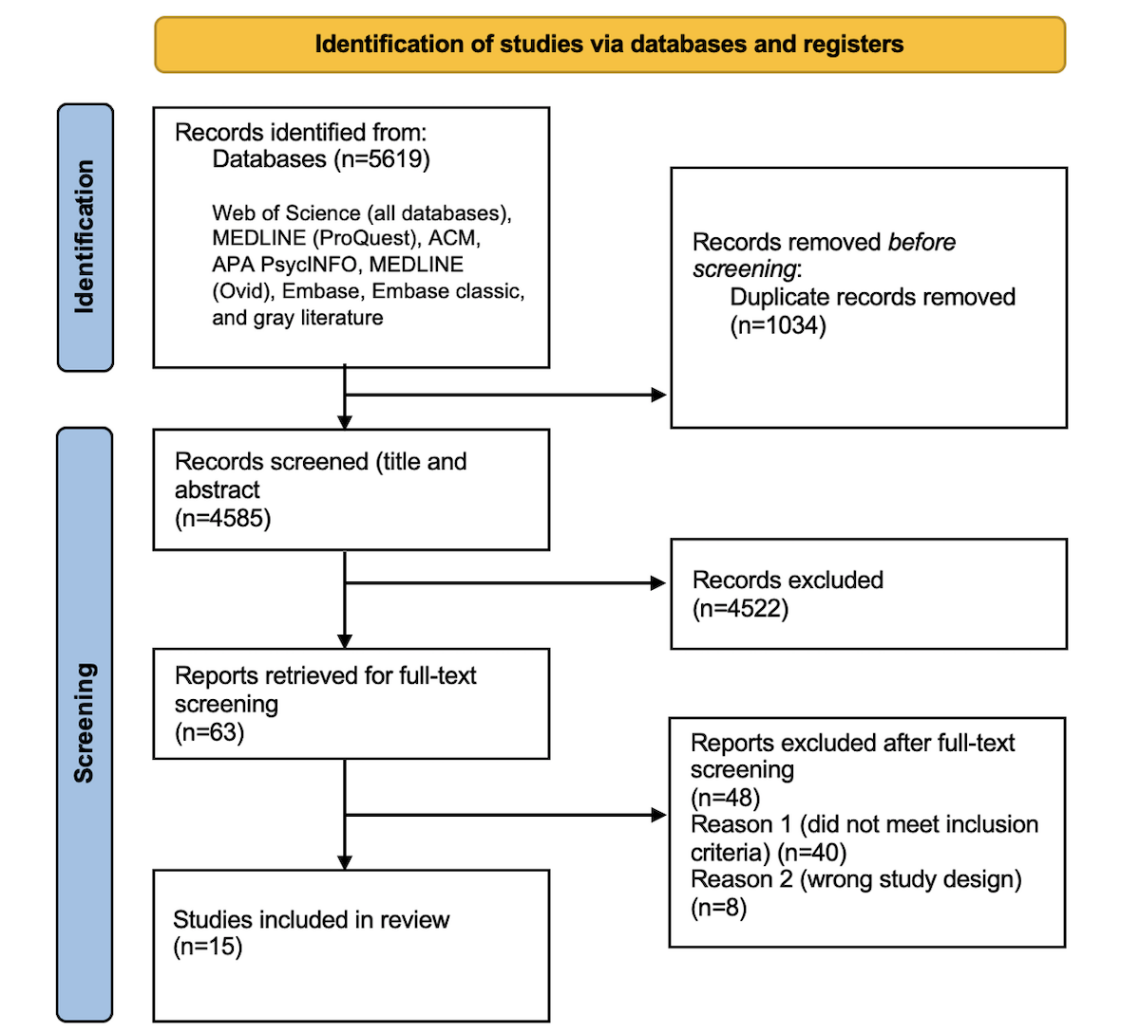
PRISMA (Preferred Reporting Items for Systematic Reviews and Meta-Analyses) 2020 flow diagram for new systematic reviews that included searches of databases and registers.

The search strategy identified 5619 potential studies for further analysis. After checking for duplicates, the number of eligible studies was reduced to 4585. After the topic, title, and abstract screening process, 63 studies were eligible. Full-text screening reduced this number to 15 studies for final analysis. Figure 1 [12] provides an overview of the screening process and the number of studies at each stage. The final 15 studies were published between 2016 and 2022 and were from Australia (n=1), Spain and the Netherlands (n=1), Spain (n=2), the United States (n=7), Canada (n=3), and Taiwan (n=1). The studies included involved the following chronic diseases: lower back pain (n=1), cardiovascular disease (n=2), hemophilia (n=1), colorectal cancer (n=1), breast cancer (n=1), stroke (n=2), type 2 diabetes (n=2), inflammatory arthritis (n=1), kidney disease (n=3), and serious mental illness (n=1).

The characteristics (author, country, topic area, number of participants, mean age of participants, chronic diseases studied, activity tracker, study duration, and study design) of the 15 studies are reported in Table 2. A context summary of the activity tracker use in each study is reported in Table 3.

**Table 2:** Characteristics of studies.

Study number	Author and year of study	Country	Topic area	Participants, n	Age of participants (years), mean (SD)	Chronic disease studied	Activity tracker	Study duration	Study design
1	Amorim et al [16], 2019	Australia	Physical activity	60	58.4 (13.4)	Chronic lower back pain	Fitbit (model not reported)	6 months	Randomized controlled trial
2	Broers et al [17], 2020	Spain and the Netherlands	Physical activity	150	61.1 (9.6)	Cardiovascular disease	Fitbit Charge HR	6 months	Randomized controlled trial
3	Carrasco et al [18], 2019	Spain	Physical activity	26	37.2 (11.1)	Hemophilia	Fitbit Flex	13 weeks	Observational study
4	van Blarigan et al [19], 2019	United States	Physical activity	42	54.0 (8.9)	Colorectal cancer	Fitbit Flex	12 weeks	Randomized controlled trial
5	Grau-Pellicer et al [20], 2020	Spain	Physical activity	41	64.6 (11.9)	Stroke	Fitlab	8 weeks	Randomized unblinded trial
6	Jiwani et al [21], 2022	United States	Physical activity	20	67.8 (5.3)	Type 2 diabetes	Fitbit (model not reported)	6 months	Single-arm study
7	Li et al [22], 2020	Canada	Physical activity	118	53.5 (14.7)	Rheumatoid arthritis	Fitbit Flex 2	27 weeks	Randomized controlled trial
8	Li et al [23], 2020	Taiwan	Physical activity	60	51.2 (11.0)	Kidney disease	Heart rate smart wristband (make and model not reported)	90 days	Randomized controlled trial
9	McNeil et al [9], 2022	Canada	Physical activity	45	51.2 (9.0)	Breast cancer	Polar M400 and A360	12 weeks	Randomized controlled trial
10	Naslund et al [24], 2016	United States	Physical activity	34	50.2 (11.0)	Mental illness	Fitbit Zip	6 months	Exploratory study
11	O’Brien et al [25], 2020	United States	Physical activity	60	65.7 (4.9)	Kidney disease	Fitbit Charge 2	6 months	Pilot feasibility study
12	Park et al [26], 2021	United States	Physical activity	60	68.0 (9.3)	Cardiac rehabilitation	Fitbit Charge 2	3 months	Randomized controlled trial
13	Sheshadri et al [27], 2020	United States	Physical activity	60	58.0 (6.3)	Kidney disease	Pedometer (make and model not reported)	6 months	Randomized controlled trial
14	Wang et al [28], 2018	United States	Physical activity	26	56.4 (7.5)	Type 2 diabetes	Pedometer (make and model not reported)	6 months	Pilot comparative trial
15	Ezeugwu et al [29], 2018	Canada	Sedentary time and physical activity	34	64.6 (12.5)	Stroke	Misfit Flash	8 weeks	Single-group intervention study

**Table 3:** Context summary of activity tracker use in studies.

Study number	Author and year of study	Summary of activity tracker use in the study
1	Amorim et al [16], 2019	The intervention group received a physical activity information booklet plus 1 face-to-face and 12 telephone-based health coaching sessions. The intervention was supported by a web-based application and an activity tracker (Fitbit).
2	Broers et al [17], 2020	Participants were provided with information, during a cardiac outpatient visit, on how to use a Fitbit activity tracker, Beddit 3 sleep monitoring tracker, and CarePortal-linked blood pressure monitor.
3	Carrasco et al [18], 2019	This study aimed to monitor daily physical activity and analyze its evolution over time in a cohort of persons with hemophilia using a commercial activity tracker. In addition, the relationship between physical activity levels, demographics, and joint health status, as well as the acceptance and adherence to the activity tracker were measured.
4	van Blarigan et al [19], 2019	Participants who had completed curative-intent treatment for colorectal cancer completed a 3-month physical activity intervention using a Fitbit activity tracker and daily SMS text messages.
5	Grau-Pellicer et al [20], 2020	Chronic stroke survivors were randomized into an intervention group and a control group. Participants in the intervention group were engaged in the multimodal rehabilitation program that consisted of supervising adherence to physical activity through a mobile health app and participating in an 8-week rehabilitation program that included aerobic, task-oriented, balance, and stretching exercises. The control group received a conventional rehabilitation program. Participants’ physical activity was measured using a Fitlab activity tracker.
6	Jiwani et al [21], 2022	Overweight older adults with self-reported type 2 diabetes were provided with a Fitbit activity tracker for self-monitoring of diet and physical activity. Additionally, they attended weight management sessions.
7	Li et al [22], 2020	Participants with rheumatoid arthritis or systemic lupus erythematosus received education and counseling from a physical therapist, used a Fitbit and a web application to obtain feedback about their physical activity, and received 4 follow-up calls from the physical therapist.
8	Li et al [23], 2020	Participants with chronic kidney disease were enrolled in the intervention group and control group. All participants were provided with wearable devices (make and model not reported) that collected exercise-related data. All participants maintained dietary diaries using a smartphone app. All dietary and exercise information was then uploaded to a health management platform. Suggestions about diet and exercise were provided to the intervention group only, and a social media group was created to inspire the participants in the intervention group.
9	McNeil et al [9], 2022	Participants were randomized to a 12-week, home-based, lower or higher intensity physical activity intervention or no intervention control group. Both intervention groups received a Polar A360 activity tracker. Study outcomes were assessed on a weekly basis with the activity tracker and included relative adherence to the prescribed physical activity.
10	Naslund et al [24], 2016	Participants diagnosed with a serious mental health disorder were enrolled in a physical activity intervention. The behavioral change program was supported by the use of a Fitbit activity monitor for measuring daily steps.
11	O’Brien et al [25], 2020	Participants diagnosed with chronic kidney disease were enrolled in a feasibility study that incorporated the use of a behavioral change application and Fitbit activity tracker to help achieve daily step goals.
12	Park et al [26], 2021	During the final week of outpatient cardiac rehabilitation, participants were randomized to an intervention group or usual care. The intervention group downloaded a motivational mobile app, received supportive push-through messages on motivation and educational messages related to cardiovascular disease management, and wore a Fitbit activity tracker to track step counts. Participants in the usual care group wore a pedometer and recorded their daily steps in a diary.
13	Sheshadri et al [27], 2020	The intervention consisted of providing participants with pedometers in conjunction with weekly semiscripted counseling sessions in which a member of the study team called the participant. Participants were asked to wear their pedometers each day and record their step counts. During the weekly counseling session, participants reported their step counts, and research personnel provided specific step goals for the upcoming week and advised about ways to incorporate more walking into the participant’s daily routine.
14	Wang et al [28], 2018	Participants were provided with a Fitbit to collect physical activity data. Using the tracker, participants were able to self-monitor their physical activity. The tracker provided the user with instant feedback. Participants were also provided with 3 daily SMS text messages that prompted physical activity.
15	Ezeugwu et al [29], 2018	Participants were provided with a sedentary behavior and physical activity behavioral change intervention. This was supported by a motivational Misfit activity tracker.

### Participants Demographics

In total, 844 adult participants took part in the 15 studies. Of these, 412 (48.8%) were male, and 432 (51.2%) were female. The mean age of all participants was 58.3 (SD 8.6) years.

### Percentage Reporting Across RE-AIM Dimensions

The RE-AIM dimensions are supported by 28 indicators. Table 4 shows the mean total number of indicators reported across the 5 main dimensions.

**Table 4:** Mean number and percentage of RE-AIM^a^ indicators reported across each dimension.

RE-AIM dimension	Mean number of indicators reported (n=15), n (%)
Reach	10 (67)
Effectiveness	10 (67)
Adoption	0.5 (3)
Implementation	9 (60)
Maintenance	2 (13)

^a^RE-AIM: Reach, Effectiveness, Adoption, Implementation, and Maintenance.

### Percentage Reporting Across the RE-AIM Indicators

#### Reach

The reach dimension is supported by 7 indicators, and the overall numbers and percentages reported are shown in Table 5.

**Table 5:** Overall number and percentage of reach indicators reported.

Reach indicator	Studies reported (n=15), n (%)
Method used to identify the target population	14 (93)
Inclusion criteria	15 (100)
Exclusion criteria	14 (93)
Use of qualitative methods to understand reach or recruitment	0 (0)
Sample size	15 (100)
Participation rate	14 (93)
Sample representatives	0 (0)

#### Effectiveness

The effectiveness dimension was supported by 6 indicators, and the overall numbers and percentages reported are shown in Table 6.

**Table 6:** Overall number and percentage of effectiveness indicators reported.

Effectiveness indicator	Studies reported (n=15), n (%)
Assessment of the effect on outcomes at the shortest assessment point	15 (100)
Imputation procedures reported (how missing data are processed)	2 (12)
The presence of quality-of-life follow-up	8 (53)
Effects at the longest follow-up	15 (100)
Use of qualitative methods to understand outcomes	2 (12)
Patient attrition or dropout rate	15 (100)

#### Adoption

The adoption dimension was supported by 6 indicators, and the overall numbers and percentages reported are shown in Table 7.

**Table 7:** Overall number and percentage of adoption indicators reported.

Adoption indicator	Studies reported (n=15), n (%)
Method of identifying target agent	0 (0)
Level of expertise of delivery agent	2 (12)
Inclusion and exclusion criteria for target agent	0 (0)
The adoption rate	1 (7)
Comparison of settings or participants of adoption versus nonadoption settings	0 (0)
Use of qualitative methods to understand either adoption at the setting level or staff participation	0 (0)

#### Implementation

The implementation dimension was supported by 5 indicators, and the overall numbers and percentages reported are shown in Table 8.

**Table 8:** Overall number and percentage of implementation indicators reported.

Implementation indicator	Studies reported (n=15), n (%)
The intervention type (individual component vs multicomponent)	15 (100)
Intensity (components of intervention)	15 (100)
The extent the protocol was delivered as intended	15 (100)
A measure of cost	0 (0)
Use of qualitative methods to understand the implementation of the study	1 (7)

#### Maintenance

The maintenance dimension was supported by 4 indicators, and the overall numbers and percentages reported are shown in Table 9.

**Table 9:** Overall number and percentage of maintenance indicators reported.

Maintenance indicator	Studies reported (n=15), n (%)
Was an individual’s behavior assessed at least 6 months following completion of the intervention	8 (53)
Is the program still in place	0 (0)
Was the program modified	1 (7)
Use of qualitative methods to understand the long-term effects	0 (0)

## Discussion

### Principal Findings

This integrative systematic review sets out to measure the level of reporting for each of the 28 RE-AIM indicators in studies identified during the search and screening phase. To the best of the lead author’s knowledge, no similar studies have been undertaken, and it is hoped these findings will be informative in the planning and evaluation of future public health interventions to support the management of chronic diseases. In summary, this review found that the RE-AIM dimensions of adoption and maintenance were underreported. The analysis of the indicators found that the use of qualitative methods to understand each dimension was underreported.

The use of commercial activity trackers within physical activity interventions either on their own or when combined with behavior change strategies has been shown to increase activity levels and reduce sedentary behavior in people diagnosed with a chronic disease [8]. In 2020, Franssen et al [8] in a systematic review identified 35 studies covering this area of research. The use of activity trackers specifically within clinical practice has been researched less. The search strategy undertaken for this review identified only 15 studies with all targeting physical activity and 1 targeting sedentary behavior. This indicates that their use in clinical practice could be increased and has a significant positive impact on the health of patients diagnosed with a chronic disease. Understanding of the reach, effectiveness, adoption, implementation, and maintenance of these interventions is vital for future programs. Using the RE-AIM framework allows for the analysis of individual studies and identifies specific areas of strength and weakness in interventions [13]. Each dimension of the RE-AIM framework in relation to the 15 identified studies is discussed in more detail in the following sections.

### Reach

Reach is set at an individual level and measures participation factors such as number, proportion, and representativeness of those taking part in an intervention [13]. RE-AIM research has shown that the majority of health care studies report sample size and proportion of participants who are willing to take part [30]. On the other hand, few studies report if the participant sample is representative of a particular patient population [31]. Of the 15 studies identified in this review, 93% (n=14) to 100% (n=15) reported the method used to identify the target population (n=14, 93%), inclusion criteria (n=15, 100%), exclusion criteria (n=14, 93%), sample size (n=15, 100%), and participation rate (n=14, 93%). Representativeness and use of qualitative methods to understand reach were not reported in any of the studies. Understanding the needs and overall demographic makeup of a population is vital to the success of an intervention, especially when dealing with technology-based programs. Issues such as age, socioeconomic background, ethnicity (language), and employment status may act as barriers for patients to engage in eHealth interventions and as such should be considered in the planning stage of a study [32]. Qualitative methods to understand a program’s reach and in particular representativeness should be incorporated during the planning stage. This can aid the researcher’s understanding of why certain patients do or do not engage in a study at the recruitment stage.

### Effectiveness

The effectiveness dimension aims to understand the impact of an intervention including outcomes, potential negative effects, wider issues such as quality of life, and variation outcomes between subgroups. These factors are vital when moving from a research environment into a real-world setting [13]. In the 15 studies under review, the effectiveness indicators assessment of effect at the shortest and longest time points and patient attrition rate were reported in all papers. Reported to a lower degree were the indicators’ imputation procedures (n=2, 12%), presence of quality-of-life measures (n=8, 53%), and use of qualitative methods to understand outcomes (n=2, 12%). The lack of reported imputation procedures increases the chance of introducing bias and impacts the internal reliability of the study. Statistical methods, such as listwise deletion, dealing with missing data should be reported especially if interventions are to be applied in economic real-world environments [30]. Positive or negative impacts of an intervention on a patient’s quality of life should also be considered in health-related studies. Focusing on just the physiological outcomes does not give a full indication of the effectiveness of an intervention. Factors such as mental and social well-being should also be considered. Qualitative methods to understand outcomes were also limited with only 2 studies reporting use. Qualitative methods can complement the quantitative data in health studies by exploring the psychological behavior change element of a patient’s participation as well as their experience of the intervention overall [33].

### Adoption

The adoption dimension moves away from the individual participant level and focuses on the settings and intervention agents. In particular, this refers to where the intervention was delivered and by whom [13]. Studies identified within this review were all undertaken within outpatient clinical care. The interventions were either delivered by the research team or health care professionals. Adoption is seldom reported in health-related studies, and this creates issues when the impact of an intervention is assessed or applied in a real-world setting. When adoption is fully reported and considered at the planning stage of a study, there is evidence that these interventions are more successful [34]. Making comparisons between settings, sites, and the teams delivering an intervention (differential adoption) can help understand good practice [34]. The 15 studies identified during this review found low levels of reporting across the 6 indicators. In fact, only the indicators’ level of expertise of the delivery agent (n=2, 12%) and adoption rate (n=1, 7%) were reported. Without this information, it is difficult to apply the methods studied in full clinical practice.

### Implementation

The implementation dimension is set at the settings level and measures how reliable and committed the delivery agent was in applying the intervention as intended along with reported monetary cost and staff time [13]. Implementation is important as it provides details of why an intervention was changed from that initially proposed. The cost factor is an important consideration for organizations intending on implementing any intervention, and this needs to be considered against the interventions’ benefits. This dimension is generally not well reported in health behavior studies [13]. This review found that implementation was overall well reported in 3 out of the 5 indicators recorded in the identified studies. The indicators’ measure of cost (n=0, 0%) and use of qualitative methods to understand the implementation of the study (n=1, 7%) were poorly reported. As these studies were delivered in outpatient clinical care, a measure of cost would be required by health care management in a real-world setting. The use of qualitative methods would also provide a greater understanding of the implementation process and how staff perceived the intervention. This would help understand the training needs of staff and any requirements to amend the intervention.

### Maintenance

The maintenance dimension is set at both the setting and individual levels. At the setting level, it refers to how well an intervention becomes part of routine health care. At an individual level, it is a measure of the long-term impact on the patient’s health. As most interventions are tested over a relatively short timescale, this is seldom reported in studies [13]. The RE-AIM evaluation framework identifies studies at or beyond 6 months as a measure of maintenance [35]. This review found that 53% (n=8) of study interventions were undertaken over 6 months or less. None are still in existence as far as the research team is aware. Only 1 study reported a modification to the intervention. None used qualitative methods to understand the long-term effect of the intervention. Stronger reporting across all 5 dimensions would help to understand why these interventions are no longer in operation. The use of qualitative methods for the maintenance dimension would have assisted in this understanding. Seven of the reviewed studies were either pilot or exploratory in design. By their very nature, these would only be planned over a short timescale.

### Study Strengths and Limitations

After the initial search, all papers were double-screened at the initial title and abstract stage and full-text stage. The review was undertaken in accordance with the PRISMA 2020 statement [12].

The initial study protocol sets out to identify specific adult age categories within each of the identified studies, for example, 18-20 and 21-30 years. As these were not reported, the protocol was amended with the focus being on adults aged 18 years and older. The lead author conducted all of the data extraction with 40% (n=6) of papers being quality-checked by 3 of the coauthors.

### Conclusions

This integrated systematic review sets out to comprehensively explore the reach, effectiveness, adoption, implementation, and maintenance of using activity trackers in clinical care to support physical activity or reduce sedentary behavior in adults diagnosed with chronic diseases. Applying the RE-AIM framework over the 5 main dimensions and 28 indicators identified a number of areas that were both well and poorly reported in studies. For this area of research, improved measurement and reporting of the dimensions adoption and maintenance are required with a focus on the settings element within studies. At an indicator level, the main area of underreporting and use was that of qualitative methods. Such methods would allow for detailed exploration of the experiences of both staff and patients at each stage of an intervention and over all RE-AIM dimensions. This would provide a better understanding of the use of activity trackers within clinical care for adults diagnosed with a chronic disease. Qualitative thematic hematic analysis involving one-to-one interviews or focus groups conducted at the prestudy, during the study, and poststudy stages with health care staff and participants would provide a greater understanding of the strengths and weaknesses of an intervention. The RE-AIM framework and findings from this systematic review also provide an insight into how such interventions could be applied in a clinical setting focusing on the dimensions of reach, effectiveness, adoption, implementation, and maintenance. The limited reporting of adoption and maintenance in studies makes it difficult to progress from a research study setting into the real-world health environment. Better reporting of these dimensions in studies would help clinicians in using activity trackers during patient care.

## Appendix

Multimedia Appendix 1 RE-AIM (Reach, Effectiveness, Adoption, Implementation, and Maintenance) data extraction Microsoft Excel spreadsheet.

Multimedia Appendix 2 PRISMA (Preferred Reporting Items for Systematic Reviews and Meta-Analysis) checklist.

## Abbreviations

PICOS: population, intervention type, comparisons, outcomes of interest, and study type
PRISMA: Preferred Reporting Items for Systematic Reviews and Meta-Analyses
RE-AIM: Reach, Effectiveness, Adoption, Implementation and Maintenance
SPIDER: sample, phenomenon of interest, study design, evaluation, and research type

## Data Availability

All data generated or analyzed during this study are included in this published paper via Multimedia Appendix 1.
